# Repurposed Drugs and Efflux Pump Inhibitors Against Gram-Negative Urinary Tract Pathogenic Bacteria

**DOI:** 10.3390/antibiotics14100988

**Published:** 2025-10-02

**Authors:** Annamária Kincses, Márta Nové, Jina Asefi, Gabriella Spengler

**Affiliations:** 1 Department of Medical Microbiology, Albert Szent-Györgyi Health Center and Albert Szent-Györgyi Medical School, University of Szeged, Semmelweis utca 6, 6725 Szeged, Hungary; kincses.annamaria90@gmail.com (A.K.); bozoki-nove.marta@med.u-szeged.hu (M.N.); jina.asefi@yahoo.de (J.A.); 2 Institute of Pharmacognosy, Faculty of Pharmacy, University of Szeged, Eötvös utca 6, 6720 Szeged, Hungary

**Keywords:** drug repurposing, bacterial efflux pump, biofilm, pH

## Abstract

**Background/Objectives**: Urinary tract infections (UTIs) represent a major healthcare challenge due to antimicrobial resistance and biofilm formation. Our aim was to evaluate whether repurposed drugs and efflux pump inhibitors (EPIs) could provide alternative strategies by investigating their antibacterial, anti-biofilm, and resistance-modifying properties against Gram-negative uropathogens under varying pH conditions. **Methods**: Clinical isolates of *Escherichia coli*, *Klebsiella pneumoniae*, and *Proteus mirabilis* were tested. Minimum inhibitory concentrations (MICs) of thioridazine (TZ), promethazine (PMZ), fluoxetine (Fx), sertraline (Sr), phenylalanine arginine β-naphthylamide (PAβN), carbonyl cyanide m-chlorophenyl hydrazone (CCCP), and the glutamine uptake inhibitor V9302 were determined at pH 5–8. Biofilm inhibition was assessed by crystal violet staining, while MIC reduction assays tested antibiotic combinations. Efflux pump inhibition was examined using an ethidium bromide accumulation assay. **Results**: TZ reduced biofilm formation in sensitive *K. pneumoniae* at all pH levels and enhanced ciprofloxacin (CIP) activity, whereas PMZ showed a weaker effect, limited mainly to neutral pH. Fx and Sr exhibited pH-dependent anti-biofilm activity, with Fx particularly effective against *P. mirabilis* at alkaline pH. PAβN consistently decreased biofilm biomass in both sensitive and resistant *K. pneumoniae* and, at pH 7–8, potentiated CIP activity with a 16-fold MIC reduction in the sensitive strain. CCCP showed pH-dependent activity, with stronger effects under acidic conditions, notably in *E. coli* and *P. mirabilis*. V9302 was a potent biofilm inhibitor in *K. pneumoniae* and resistant *E. coli* and interfered with efflux activity, showing strong effects in acidic environments. **Conclusions**: Repurposed drugs and EPIs may be useful as antibiotic adjuvants or biofilm inhibitors in treating resistant UTIs.

## 1. Introduction

Drug repurposing has emerged as a promising strategy to combat urinary tract infections (UTIs) caused by pathogenic bacteria, particularly in the context of catheter-associated urinary tract infections (CAUTIs). The emergence of antibiotic resistance represents a major challenge in the treatment of UTIs, and efflux pump (EP) overexpression plays an important role in the development of multidrug resistance (MDR) in Gram-negative uropathogens. A key mechanism is the reduction in intracellular drug concentrations, either by limiting uptake or by actively pumping antibiotics out of the cell [1]. Fluoroquinolone resistance in *Escherichia coli* has been associated with the overexpression of EP-related genes, including structural components (*acrA*, *mdfA*, *yhiU*, and *yhiV*) and regulatory factors (such as *marA*) [2,3]. PCR revealed that *tolC* and *ynfA* EP genes were highly expressed in 75–80% of multidrug-resistant *E. coli* isolates from UTI patients, highlighting their major role in resistance [4]. In *E. coli* and *Klebsiella pneumoniae*, RND efflux systems such as AcrAB-TolC, AcrAD-TolC, and AcrFE-TolC are common, and Al-Dahmoshi et al. found that over 90% of isolates were MDR, especially against β-lactams and cephalosporins [5]. Fluoroquinolone resistance in *K. pneumoniae* is mainly explained by mutations in DNA gyrase and topoisomerase IV, but reduced susceptibility is further supported by plasmid-mediated quinolone resistance determinants, including QepA, and OqxAB EPs. Importantly, the OqxAB system has been shown to help *K. pneumoniae* tolerate increasing concentrations of ciprofloxacin [6]. Taken together, these findings emphasize the clinical importance of EPs in UTI pathogens and highlight their potential as targets for adjuvant strategies.

Drug repurposing, also known as drug repositioning, serves as a strategic approach to identify new therapeutic uses for existing drugs. Recent studies have focused on repurposing antidepressants such as sertraline (Sr), duloxetine, and fluoxetine (Fx), which are selective serotonin reuptake inhibitors (SSRIs), demonstrating their potential antibacterial and anti-biofilm activities against common uropathogens like *E. coli* and *Enterococcus faecalis* [7,8,9,10,11]. In addition, all three drugs significantly inhibited biofilm formation, a critical factor in the persistence of CAUTIs. The studies suggest that these antidepressants can be effective in preventing bacterial colonization on catheter surfaces, which is essential for reducing infection rates. Sr [12] and Fx [13] have also been shown to possess anti-biofilm activity against UTI causing bacteria.

The antipsychotic phenothiazine-type drugs have been described as potent antibacterial compounds [14]. Thioridazine (TZ) and promethazine (PMZ), both members of the phenothiazine class, have been identified as inhibitors of bacterial EPs. By inhibiting efflux pump activity, these compounds can enhance the efficacy of conventional antibiotics against resistant bacterial strains. Furthermore, it was suggested that phenothiazines like TZ and PMZ possess structural attributes that enable direct interaction with EPs. Such interactions may potentially alter the proton motive force (PMF), rendering it less favorable for the pumps to expel antibiotics.

The pH level of the urine is widely varied (pH 4.5–8) and can easily be modified by diet or medication. Since pH is an essential factor in colonization and proliferation of urinary pathogenic bacteria, it affects the effectiveness of antibiotics and may play an important role in the treatment and prevention of UTI [15]. Certain antibiotics perform better at specific pH levels. For instance, fluoroquinolones and aminoglycosides are more effective in alkaline conditions, while fosfomycin and nitrofurantoin thrive in acidic environments [16].

In the present study, resistance modifier and efflux pump inhibitor (EPI) molecules were investigated on six UTI bacterial strains of *K. pneumoniae*, *Proteus mirabilis*, and *E. coli* previously characterized regarding their phenotypical and genetic resistance pattern [17]. The activity of EPIs was assessed at pH 5, 6, 7, and 8, and their anti-biofilm effects were evaluated using crystal violet staining under the same pH conditions. The most effective EPIs were combined with ciprofloxacin (CIP) and fosfomycin tromethamine (FO) antibiotics and the reduction in the minimum inhibitory concentration (MIC) of antibiotics were determined at different pHs.

## 2. Results

### 2.1. Antibacterial Activity

PMZ had no MIC values on the clinical strains investigated in this study at pH 5, 6, 7, and 8 (MIC > 100 µM). The antibacterial activity of TZ was similar to the one of PMZ: on the sensitive *E. coli* 33504 strain, TZ exerted an MIC of 100 µM at all pHs; however, the resistant *E. coli* 32313 was more sensitive towards TZ at the neutral and alkaline pHs (MIC of 100 µM). On the *K. pneumoniae* 33443, 33163 and on the *P. mirabilis* 33877, 32470 strains, the MIC value was more than 100 µM at all pHs (Table 1).

The SSRI drug Fx had no antibacterial activity on the strains investigated; however, the other SSRI compound Sr was moderately active on the *E. coli* strains. At pH 5 and 6, the MIC was 100 µM on the sensitive *E. coli* 33504 strain; however, Sr showed an MIC of 50 µM at pH 7 and 8. Regarding the resistant *E. coli* 32313 strain, Sr was active only at pH 8 exhibiting an MIC of 50 µM (Table 1).

The well-known efflux pump inhibitor phenylalanine arginine β-naphthylamide (PAβN) had no antibacterial activity on the strains investigated at four pHs. The protonophore compound carbonyl cyanide m-chlorophenyl hydrazone (CCCP) had slight activity only at pH 5 on *K. pneumoniae* strains (MIC of 25 µM on the sensitive strain and MIC of 100 µM on the resistant strain). CCCP was very active on *P. mirabilis* strains: on the sensitive strain the MIC was 6.25 µM at pH 5 and pH 6, 12.5 µM at pH 7 and 25 µM at pH 8. Furthermore, CCCP exerted antibacterial activity on the resistant *P. mirabilis* 32470 strain: the MIC was 25 µM at all pHs. The glutamine metabolism inhibitor V9302 had no activity on the *K. pneumoniae* strains. In addition, it showed moderate and slight activity on the sensitive and resistant *P. mirabilis* strains at pH 7, respectively (MIC of 50 µM and 100 µM). On the *E. coli* strains, the best antimicrobial activity was demonstrated at pH 6 and pH 7 on the sensitive strain (MIC of 50 µM at both pHs) and at pH 5, 6, and 7 on the resistant strain (MIC of 100 µM at three pHs; Table 2).

### 2.2. Anti-Biofilm Activity

**Figure 1:**
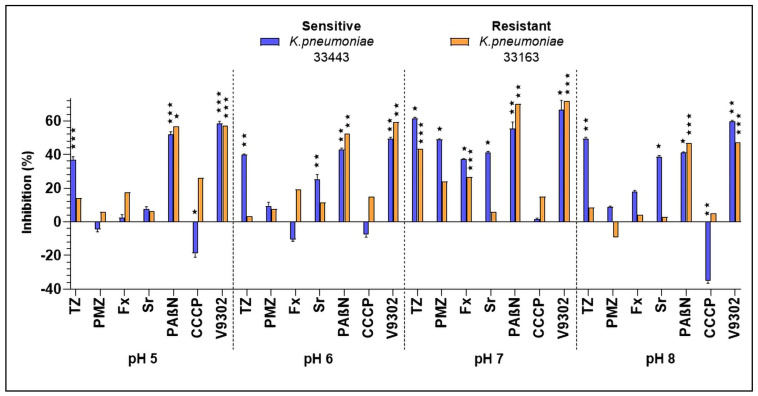
Biofilm inhibition exerted by the efflux pump inhibitor (EPI) compounds on the *K. pneumoniae* strains. The levels of significance were ⋆ *p* < 0.05, ⋆⋆ *p* < 0.01, and ⋆⋆⋆ *p* < 0.001. TZ: thioridazine, PMZ: promethazine, Fx: fluoxetine, Sr: sertraline, PAβN: phenylalanine-arginine β-naphthylamide, CCCP: carbonyl cyanide m-chlorophenyl hydrazone.

The biofilm inhibiting activity of the compounds depends on the pH of the environment. The inhibition of bacterial biofilm was tested either at 100 µM if the compound had no MIC (>100 µM) or at MIC/2 if the compound has an MIC. Based on our results, the compounds showed pH-dependent inhibition. At pH 5, the most potent anti-biofilm agents were V9302, PAβN, and TZ demonstrating the inhibition of bacterial biofilms by 58.43%, 52.14%, and 36.97%, respectively, on the sensitive *K. pneumoniae* 33443 strain. At pH 6, the above-mentioned derivatives showed the same inhibition pattern; however, at pH 6, Sr exerted an anti-biofilm activity of 25.22% on the sensitive *K. pneumoniae* 33443 strain. On the resistant *K. pneumoniae* 33163 strain the most active anti-biofilm drug was V9302 (inhibition: 57.36%) at pH 5, followed by PAβN (inhibition: 56.71%), CCCP (inhibition: 26.03%), and Fx (inhibition: 17.73%), furthermore, at pH 6, similar tendency of inhibition was observed (V9302: 59.4%; PAβN: 52.34%, Fx: 19.14%, CCCP: 14.9%). At pH 7, remarkable biofilm inhibition was measured in the presence of phenothiazines on the sensitive *K. pneumoniae* 33443 strain: 61.51% and 49.05% inhibition by TZ and PMZ, respectively. On the resistant *K. pneumoniae* 33163 strain, lower inhibition rate was detected: 43.55% for TZ and 23.79% for PMZ. On the resistant *K. pneumoniae* strain, the EPI compound PAβN showed more active inhibition (70.31%) than on the sensitive strain (55.45%). The SSRIs Sr and Fx inhibited biofilm formation by 41.01% and 37.38% on the sensitive *K. pneumoniae* 33443 strain at pH 7, respectively. Interestingly, Sr had no effect on the resistant *K. pneumoniae* 33136 strain and Fx showed an inhibition of 26.78% on this strain. Surprisingly, V9302 was the most potent anti-biofilm agent at pH 7 on both *K. pneumoniae* strains showing more activity on the resistant strain (71.88%) compared to the sensitive one (66.59%). At pH 8, TZ was more potent on the sensitive *K. pneumoniae* strain (49.47% inhibition) than on the resistant one (8.51% inhibition). The sensitive *K. pneumoniae* strain was more susceptible towards anti-biofilm agents at pH 8: Sr and PAβN exerted an activity about 40%, furthermore, the most active derivative was V9302 (inhibition: 59.87%). On the resistant *K. pneumoniae* 33136 strain, only two drugs, V9302 and PAβN had efficacy (inhibition of 47.21% and 46.71%, respectively; Figure 1).

**Figure 2:**
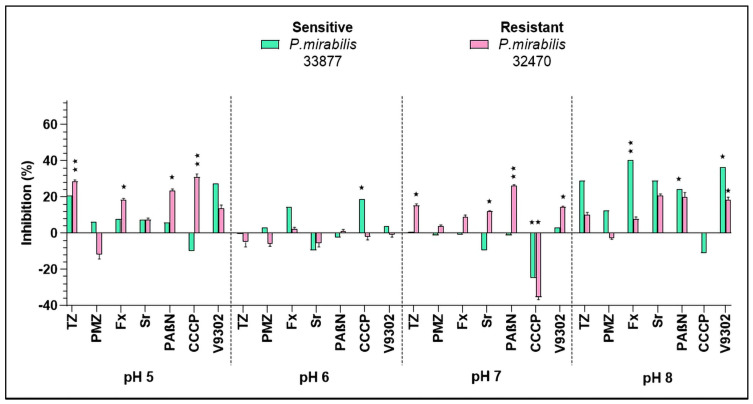
Biofilm inhibition exerted by the EPI compounds on the *P. mirabilis* strains. The levels of significance were ⋆ *p* < 0.05 and ⋆⋆ *p* < 0.01. TZ: thioridazine, PMZ: promethazine, Fx: fluoxetine, Sr: sertraline, PAβN: phenylalanine-arginine β-naphthylamide, CCCP: carbonyl cyanide m-chlorophenyl hydrazone.

The isolates of *P. mirabilis* were less sensitive towards the tested anti-biofilm drugs. TZ was active at pH 5 and pH 8, showing less activity at the acidic pH (inhibition of 20.92%) compared to the alkaline pH (inhibition of 28.95%) on the sensitive *P. mirabilis* 33877 strain. Fx and Sr exerted anti-biofilm activity at pH 8 (40.37% and 29.08%, respectively); furthermore, the EPI compound PAβN could also interfere with biofilm formation (24.48% of inhibition) on the sensitive *P. mirabilis* 33877 strain. Out of the tested resistance modifiers, only PAβN showed efficacy at three pHs (pH 5, 7, and 8) on the resistant *P. mirabilis* 32470 strain, but the activity was around 20% at all pHs. CCCP and TZ were active at pH 5 (inhibition of 30.98% and 28.63%, respectively) on the resistant *P. mirabilis* (Figure 2). At pH 8, CCCP promoted biofilm formation in the resistant *P. mirabilis* isolate, with a change of −103.59% (± 1.07) compared to the untreated control.

**Figure 3:**
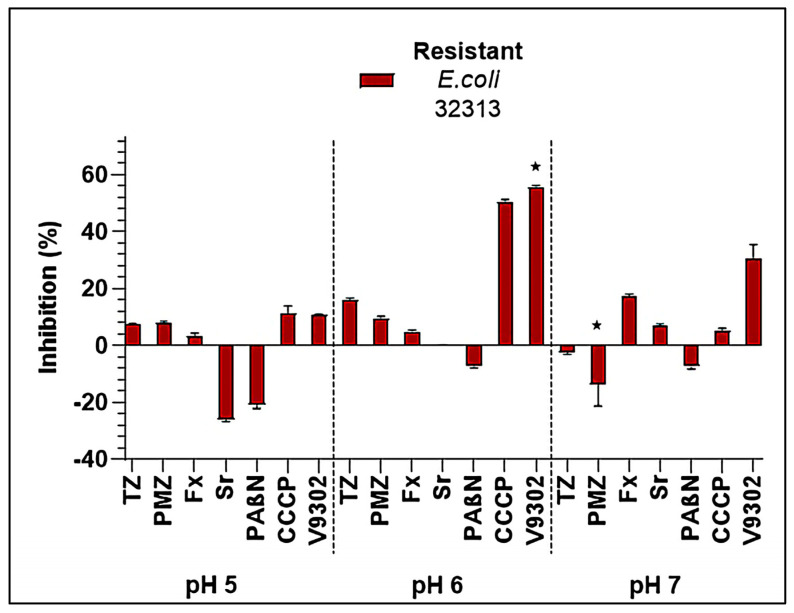
Biofilm inhibition exerted by the EPI compounds on the *E. coli* 32313 bacterial strain. The level of significance was ⋆ *p* < 0.05. TZ: thioridazine, PMZ: promethazine, Fx: fluoxetine, Sr: sertraline, PAβN: phenylalanine-arginine β-naphthylamide, CCCP: carbonyl cyanide m-chlorophenyl hydrazone.

The sensitive *E. coli* 33504 strain did not produce biofilms. The resistant 32313 strain produced biofilm at pH 5, 6, and 7. Biofilm formation was inhibited by CCCP (50.21%) and V9302 (55.61%) at pH 6. At pH 7, V9302 had less activity on this strain (30.36%) and Fx exerted a slight anti-biofilm effect (17.53%; Figure 3).

### 2.3. MIC Reduction Assay

The compounds showing 35% or more biofilm inhibiting activity were combined with either CIP or FO on the biofilm producing strains of *K. pneumoniae*, *P. mirabilis* and *E. coli*. The resistance modifiers were used at sub-inhibitory concentrations (100 µM or ½ MIC). Augmented antibacterial activity was detected for CIP on both the sensitive and resistant *K. pneumoniae* strains, and for FO in the sensitive strain. The phenothiazines PMZ and TZ could reduce the MIC of CIP by 50% at pH 7; furthermore, PAβN was active at pH 6, 7, and 8 resulting in a 4-fold, 16-fold, and 16-fold decrease in the MIC on the sensitive *K. pneumoniae* 33443 strain. On contrary, this combination was less active on the resistant *K. pneumoniae* 33163 strain because only a 2-fold decrease in the MIC of CIP was observed at pH 8. In addition, a 2-fold reduction in the MIC of FO was detected at pH 8 in the sensitive strain 33443 when combined with PAβN. The other combinations were not effective on the strains investigated in the assay. Table 3 includes only those results where an effect was observed.

### 2.4. Inhibition of Bacterial Efflux Pumps

**Figure 4:**
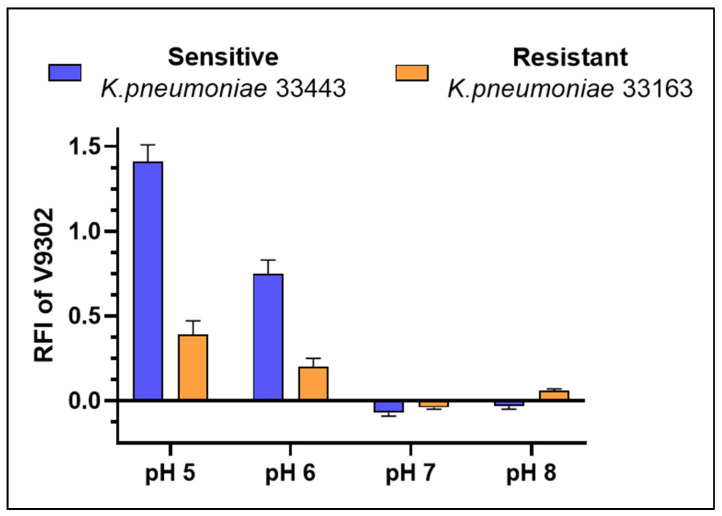
Efflux pump inhibition by V9302 based on relative fluorescence index (RFI) on the *K. pneumoniae* strains.

V9302 was specifically tested because no previous data exists regarding its potential efflux pump inhibitory activity in bacteria. The assay was restricted to *Klebsiella* strains, as this compound exhibited pronounced anti-biofilm effects against them at all four pH values. At a concentration of 100 μM, V9302 increased the EB accumulation in both *K. pneumoniae* strains at acidic pH values (pH 5 and 6). The effect was strongest at pH 5, where the relative fluorescence index (RFI) was 1.41 in sensitive 33443 strain and 0.39 in resistant 33163 strain. A moderate increase was also observed at pH 6 (RFI = 0.75 for strain 33443 and 0.20 for strain 33163). In contrast, at neutral and alkaline conditions (pH 7 and 8), V9302 had no significant effect on EB accumulation in either strain (Figure 4).

## 3. Discussion

In our study, both TZ and PMZ showed no significant antibacterial activity, with MIC values ≥100 µM in all tested UTI strains. These findings are in line with previous reports, which have demonstrated that phenothiazines lack clinically relevant intrinsic antibacterial potency. Earlier studies have shown that *E. coli* ATCC 25922 exhibited a TZ MIC of 100 mg/L [18], and *P. mirabilis* UTI strain showed MIC of 800 µg/mL [13]. Similarly, PMZ MIC values reported by Dastidar et al. were 100–200 µg/mL against Gram-negative bacteria [19], supporting our observation that therapeutic concentrations are below those required for bacterial growth inhibition.

Despite this lack of intrinsic antibacterial activity, both compounds displayed notable anti-biofilm and adjuvant effects. TZ inhibited biofilm formation of *K. pneumoniae* 33443 by 61.51% at neutral pH. Kvist et al. reported similar results, showing that TZ significantly reduced biofilm formation in a urinary tract isolate of *K. pneumoniae*, with inhibition levels of up to ~60% at 50 µg/mL [20]. In addition, TZ potentiated CIP activity, reducing the MIC by 2-fold in *K. pneumoniae* 33443 strain. These effects can be explained by the efflux pump inhibitory activity of TZ, as efflux systems in Gram-negative bacteria are known to export not only antibiotics but also metabolites and quorum-sensing molecules that contribute to biofilm maturation [21].

PMZ showed similar effects, but its activity was pH dependent. At neutral pH, PMZ inhibited the biofilm formation of *K. pneumoniae* 33443 by 49.05% and reduced the MIC of CIP by 2-fold. A strong biofilm inhibitory effect of PMZ at 100 µg/mL has also been reported in a clinical isolate of *K. pneumoniae* [22]. Importantly, the EPI properties of PMZ are pH dependent: in *E. coli* K-12 AG100, its activity was less effective at acidic pH compared to neutral conditions, as the PMF enhances the activity of the AcrAB-TolC efflux system under acidic conditions [23]. Although this finding has been described in *E. coli*, it may explain why the biofilm inhibitory and CIP-potentiating effects of PMZ were more pronounced at neutral pH in *K. pneumoniae*.

Considering these findings, our results confirm that while TZ and PMZ lack antibacterial activity, both compounds act as biofilm inhibitors and potentiate fluoroquinolone activity in *K. pneumoniae*. For PMZ, this effect was pH dependent, whereas for TZ it appears to be independent of pH, reflecting differences in their efflux pump inhibitory properties.

Both Fx and Sr, SSRIs, exhibited limited antibacterial activity in our assays, but showed notable anti-biofilm effects, suggesting their relevance lies more in biofilm modulation than in direct antibacterial activity.

Fx did not show antibacterial activity against the studied clinical isolates (MIC > 100 µM at all tested pH values), which is consistent with previous reports. MIC values have generally been high for Gram-negative bacteria, around 256 µg/mL for *E. coli*, >256 µg/mL for *K. pneumoniae*, and approximately 128–256 µg/mL for *P. mirabilis* [24]. Fx showed moderate anti-biofilm effects in *K. pneumoniae* 33443 (37% inhibition at pH 7) and in *P. mirabilis* 33877 (40% inhibition at pH 8). Comparable observations have been reported in a catheter-associated model, where Fx significantly reduced crystalline biofilm formation of *P. mirabilis*. In that system, artificial urine initially had a pH of 6.1, but urease activity rapidly increased the pH towards alkaline conditions (around 8), creating an environment favorable for crystalline biofilm development. Even under these conditions, Fx substantially decreased biofilm biomass [13], demonstrating its capacity to interfere with biofilm growth independently of bactericidal activity.

In contrast, Sr displayed moderate antibacterial activity against *E. coli*, with MIC values of ≥100 µM at acidic pH (5 and 6) and 50 µM at neutral and alkaline pH (7 and 8). No activity was observed against *K. pneumoniae* or *P. mirabilis*. Previous studies have confirmed similarly high MICs, with values of 64–128 µg/mL for *E. coli*, 128–256 µg/mL for *K. pneumoniae*, and >256 µg/mL for *Proteus* species [24]. Beyond this, Sr exhibited pH-dependent anti-biofilm effects, including 41.04% inhibition in the sensitive *K. pneumoniae* strain at pH 7 and 38.85% inhibition at pH 8, while the resistant strain remained unaffected. In *P. mirabilis*, inhibition was observed only at pH 8 (29.08%). These results are in line with a study showing that Sr significantly reduces catheter-associated *E. coli* biofilms in vitro [10], although the role of pH has not been investigated in this experiment.

The anti-biofilm effects of both Fx and Sr can be explained by efflux pump inhibition. Increased intracellular accumulation of EB has been demonstrated in *P. mirabilis* exposed to Fx, confirming impaired efflux. Similarly, Sr has been shown to interfere with the AcrAB-TolC efflux system in *E. coli* [9], reducing efflux activity and increasing intracellular drug accumulation. Since efflux pumps contribute not only to antibiotic resistance but also to biofilm maturation through the export of signaling molecules and metabolites, their inhibition is a likely explanation for the anti-biofilm activity observed. Additional mechanisms—such as membrane perturbation or interference with quorum-sensing signals—have also been proposed for SSRIs, although these remain hypothetical [24].

PAβN showed no direct antibacterial activity in our assays (MIC ≥ 100 µM), which is consistent with reports describing MIC values > 200 µg/mL for *E. coli* UTI strain 83972 and *K. pneumoniae* UTI strain i222-86 [20]. Despite this lack of effect, PAβN significantly reduced *K. pneumoniae* biofilms at all tested pH values. In the sensitive strain (33443), inhibition ranged between 40.07 and 52.14%, while in the resistant strain (33163) the strongest effect was observed at pH 7, with a reduction of 70.31%.

In strain *K. pneumoniae* 33443, PAβN reduced the MICs of CIP 4-fold at pH 6 and up to 16-fold at pH 7 and 8, while in strain 33163 a 2-fold decrease was observed at pH 8. Additionally, PAβN reduced the MIC of FO 2-fold at pH 8 in strain 33443. A possible explanation for the absence of potentiation at pH 5 in MIC reduction assays is that the PMF (high concentration of hydronium ions) reduces the efflux pump inhibitory activity of PAβN by driving effectively the efflux transporters, thereby limiting the impact of the inhibitor on antibiotic susceptibility. In contrast, biofilm assays—performed over longer incubation periods—still revealed inhibition even at pH 5, suggesting that time-dependent limitations of efflux activity may contribute to the anti-biofilm activity under acidic conditions. Comparable results have been reported in clinical isolates, where PAβN has been effective at inducing drug sensitivity against UTI *K. pneumoniae* isolates and reduced CIP MICs by 8–64-fold [25].

PAβN was the first EPI identified against Gram-negative bacteria and is now widely regarded as the prototypical compound, serving as the most frequently applied reference EPI in this group. More recently, it has been shown to act as a competitive substrate for a broad spectrum of transporters, significantly lowering the MICs of quinolones and other AcrAB substrates in MDR *K. pneumoniae* [26].

CCCP showed pH-dependent antibacterial activity. In *P. mirabilis*, the sensitive strain 33877 was inhibited at 6.25 µM under acidic conditions (pH 5 and 6), at 12.5 µM at pH 7, and at 25 µM at pH 8, while the resistant strain 32470 showed a constant MIC of 25 µM across all pH values. In *E. coli*, both the sensitive (33504) and resistant (32313) strains displayed the lowest MICs at pH 5 (12.5 µM), with values increasing to 25 µM at pH 7 and 100 µM at pH 8. For *K. pneumoniae*, activity was limited to acidic conditions, with MICs of 25 µM in the sensitive strain (33443) and 100 µM in the resistant strain (33163) at pH 5, while no inhibition was detected at higher pH values, where the concentration of hydronium ions is low. Biofilm assays revealed inhibition in resistant *E. coli* 32313, where CCCP reduced biomass by 50.21% at pH 6, and in *K. pneumoniae* 33163, where a 26% reduction was observed at pH 5. Our study corroborates previous findings in *E. coli* MG1655, where biofilm formation has been shown to decrease significantly at sub-MIC of CCCP, with an 8 mg/L treatment reducing biomass by approximately 60% compared with untreated control [27].

CCCP is a classical protonophore that disrupts the PMF by disrupting the proton gradient across the membrane, thereby perturbing processes such as antibiotic transport and efflux activity [28]. This mechanism explains why lower MIC values were observed for *E. coli* and *P. mirabilis* at acidic pH, where proton gradients are stronger and thus more vulnerable to collapse. By depolarizing the plasma membrane and reducing ATP production, protonophores such as CCCP indirectly affect the activity of proton pumps and cellular metabolism, further contributing to growth inhibition [29].

Efflux pumps can extrude protonophores, providing partial protection, although this activity is heterogeneous within bacterial populations. This heterogeneity may help to explain why the inhibitory effects remain detectable in biofilm assays, where longer incubation periods allow cumulative activity even in subpopulations with weaker efflux capacity.

In addition to acting as a protonophore that disrupts PMF and inhibits RND-type efflux pumps, CCCP also interferes with bacterial metabolism more broadly. Therefore, it remains under debate whether its effects on biofilm formation and antibiotic resistance derive mainly from efflux pump inhibition or from alterations in central metabolic processes [30]. Altogether, the combined disruption of PMF, efflux activity, and energy metabolism provides an explanation for the observed pH-dependent effects of CCCP in both planktonic and biofilm models.

V9302 displayed no antibacterial activity against *K. pneumoniae* strains at any tested pH (MIC > 100 µM). In *P. mirabilis*, moderate inhibition was detected on the sensitive strain 33877 at pH 7 (MIC 50 µM), while the resistant strain 32470 showed only weak activity at the same pH (MIC 100 µM). In *E. coli*, V9302 was most active against the sensitive strain 33504 at pH 6 and 7 (MIC 50 µM at both), whereas the resistant strain 32313 showed a constant MIC of 100 µM at pH 5, 6 and 7. Regarding biofilm formation, V9302 was consistently one of the most potent inhibitors in *K. pneumoniae*. In the sensitive strain 33443, inhibition ranged between 50 and 67% across pH values, with the strongest reduction at pH 7 (66.59%). In the resistant strain 33163, inhibition was similarly strong, reaching its highest value of 71.88% at pH 7. In *E. coli* 32313, inhibition was 55.61% at pH 6 and 30.36% at pH 7, while in *P. mirabilis* 33877 a moderate effect (36.47%) was observed at pH 8. These data suggest that V9302 interferes with biofilm development more efficiently than with planktonic growth.

In addition, V9302 showed pH-dependent efflux pump inhibition. At 100 µM, it markedly increased EB accumulation in both *K. pneumoniae* strains at acidic pH, with the strongest effect at pH 5 (RFI 1.41 ± 0.10 in strain 33443; RFI 0.39 ± 0.08 in strain 33163). A moderate increase was also observed at pH 6, whereas no significant effect was detected at neutral or alkaline conditions (pH 7 and 8). Interestingly, while efflux inhibition was strongest at acidic pH, antibacterial activity peaked at neutral pH, indicating a divergent pH dependence of the two effects.

V9302 was originally developed as a potent small-molecule inhibitor of the glutamine transporter ASCT2 (SLC1A5) in mammalian cells, where it was shown to block glutamine uptake and suppress cancer cell growth in preclinical models [31]. It has been shown that V9302 inhibits the MDR transporter P-glycoprotein (ABCB1) [32]. To date, V9302 has not been investigated in bacterial systems; therefore, our study provides the first evidence of its effects on bacterial physiology. Considering the established mechanism in mammalian cells, a similar mode of action may be assumed in bacteria, as glutamine is also a key metabolite in prokaryotic physiology. In bacteria, glutamine functions not only as one of the standard amino acids in protein synthesis but also as a precursor for multiple nitrogen-containing compounds and as the main product of ammonium assimilation. Due to its versatile roles, glutamine metabolism is tightly regulated in response to the cellular nitrogen status [33]. The importance of glutamine extends to biofilm formation as well. Zhao et al. demonstrated that adding glutamine enhanced antibiotic uptake and killing of multidrug-resistant bacterial biofilms both in vitro and in mouse UTI models [34]. This suggests that glutamine availability modulates biofilm formation and drug susceptibility even in Gram-negative pathogens like *E. coli* and *K. pneumoniae*.

In conclusion, these observations provide a strong rationale for exploring V9302 in bacterial models: by impairing glutamine-dependent pathways, it could disrupt metabolic and structural functions—ultimately inhibiting both biofilm formation and tolerance.

## 4. Materials and Methods

### 4.1. Bacterial Strains

Clinical isolates of *E. coli* 33504, 32313; *K. pneumoniae* 33443, 33163; and *P. mirabilis* 33877, 32470 were obtained from the Institute of Clinical Microbiology, University of Szeged, and included in the present study. The species identities of these isolates had been confirmed in our previous work using both MALDI-TOF MS and conventional biochemical assays [17].

### 4.2. Determination of Minimum Inhibitory Concentrations by Microdilution Method

The MICs of tested compounds (TZ, PMZ, Fx, Sr, PAβN, CCCP, and ((2S)-2-amino-4-[bis[[2-[(3-methylphenyl)methoxy]phenyl]methyl]amino]butanoic acid (V9302)) were determined by the microdilution method in 96-well plates according to the Clinical and Laboratory Standards Institute (CLSI) guidelines [35] using Mueller-Hinton broth (MHB) at pH 5, 6, 7, and 8. The bacterial strains were cultured separately in media adjusted to pH 5–8 overnight at 37 °C, and the cultures grown at the corresponding pH were used in the assay.

### 4.3. Measuring Biofilm Formation Using Crystal Violet

The anti-biofilm activity of the tested compounds (TZ, PMZ, Fx, Sr, PAβN, CCCP, and V9302) against *E. coli*, *K. pneumoniae*, and *P. mirabilis* strains was assessed using the crystal violet (CV; 0.1% *v*/*v*) staining method, which detects the total biofilm biomass formed. Overnight cultures were diluted to an optical density (OD) of 0.1 at 600 nm in Luria–Bertani broth adjusted to pH 5, 6, 7, and 8. The bacterial strains were cultured separately in media of each pH value overnight at 37 °C, and the cultures grown at the corresponding pH were used in the assay.

To determine the baseline biofilm-forming ability, cultures were first tested without compounds at each pH, and only those conditions where the OD at 600 nm exceeded 0.5 were selected for further evaluation of the compounds. The compounds were applied at ½ MIC when measurable MIC values were available, or at 100 μM when the MIC was more than 100 μM.

For the assay, the bacterial cultures and compounds were added to 96-well microtiter plates in a final volume of 200 μL per well. The plates were incubated at 30 °C for 48 h with gentle agitation (100 rpm). After incubation, the medium was removed, and the wells were washed with tap water to eliminate unattached cells. Then, 200 μL of CV was added and incubated for 15 min at room temperature, followed by washing and solubilization of the bound dye with 200 μL of 70% ethanol. Biofilm biomass was quantified by measuring the OD at 600 nm using a Multiscan EX ELISA plate reader (Thermo Labsystems, Cheshire, WA, USA). The anti-biofilm effect of the compounds was expressed as the percentage reduction in biofilm biomass relative to the untreated control.

### 4.4. Resistance Modulation Assay

The resistance-modulating effects of the selected compounds in combination with CIP and FO were evaluated by a MIC reduction assay on *K. pneumoniae*, *P. mirabilis*, and *E. coli* strains. Briefly, CIP or FO was serially two-fold diluted in 96-well microtiter plates containing MH broth adjusted to pH 5, 6, 7, and 8, and the compounds were added at subinhibitory concentrations (100 μM or ½ MIC, depending on whether the MIC exceeded 100 μM). Only compounds that exhibited at least 35% inhibition of biofilm formation were included in this assay and they were tested exclusively at the pH values where such activity was observed.

Prior to the assay, the bacterial strains were cultured overnight in MHB adjusted to the corresponding pH (5–8) at 37 °C, and a 10^−4^ dilution of these cultures was added to each well. The final volume was 200 μL per well. The plates were incubated at 37 °C for 18 h. MIC values of antibiotics alone and in combination with the tested compounds were determined by visual inspection.

### 4.5. Real-Time Ethidium Bromide Accumulation Assay

The impact of V9302 on ethidium bromide (EB) accumulation was determined in the two *K. pneumoniae* strains by the automated EB method using a CLARIOstar Plus plate reader (BMG Labtech, Aylesbury, UK). The strains were cultured until reaching an OD of 0.6 at 600 nm. Cells were harvested by centrifugation at 13,000× *g* for 3 min, washed with phosphate-buffered saline (PBS) adjusted to pH 5, 6, 7, and 8, and resuspended in the corresponding PBS. V9302 was applied at a concentration of 100 μM to PBS containing a non-toxic concentration of EB (1 µg/mL). Subsequently, 50 μL of the EB solution containing the compound was transferred into 96-well black microtiter plates (Greiner Bio-One Hungary Kft, Mosonmagyaróvár, Hungary), followed by the addition of 50 μL of bacterial suspension (OD_600_ 0.6) into each well. The plates were then placed into the CLARIOstar reader, and fluorescence was recorded at excitation/emission wavelengths of 530/600 nm every minute for one hour in real time.

From the real-time curves, the RFI was calculated at the last time point (min 60) using the formula:

RFI = (RF_treated_ − RF_untreated_)/RF_untreated_

where RF_treated_ is the relative fluorescence (RF) in the presence of V9302, and RF_untreated_ is the RF of the solvent control (DMSO).

### 4.6. Statystical Analysis

Results are expressed as mean ± standard deviation (SD) determined for three replicates from three independent experiments. Statistical analyses were carried out using GraphPad Prism version 8.0 for Windows (GraphPad Software, San Diego, CA, USA). An unpaired Student’s *t*-test was used to compare treated and untreated samples. Statistical significance was indicated by *p* < 0.05, *p* < 0.01, and *p* < 0.001. 95% confidence intervals (CI) were calculated for datasets where statistically significant differences were observed (Table S1).

## 5. Conclusions

Our findings demonstrate that while the tested repurposed drugs and EPIs generally lacked intrinsic antibacterial activity, several compounds—including TZ, PMZ, Fx, Sr, PAβN, CCCP, and V9302—showed notable pH-dependent anti-biofilm and resistance-modifying effects against Gram-negative uropathogens. In particular, TZ and PMZ enhanced CIP efficacy in *K. pneumoniae*, PAβN strongly potentiated fluoroquinolone activity, and V9302 emerged as a novel biofilm inhibitor with efflux-modulating potential. These results highlight the therapeutic promise of drug repurposing and efflux pump inhibition as adjuvant strategies to combat biofilm-associated, multidrug-resistant urinary tract infections. Further in vivo investigations are warranted to validate their clinical applicability.

## Figures and Tables

**Table 1:** Minimum inhibitory concentrations (MICs) for thioridazine, promethazine, fluoxetine, and sertraline on *E. coli*, *K. pneumoniae*, and *P. mirabilis* strains.

MIC (µM)	Thioridazine				Promethazine				Fluoxetine				Sertraline			
	pH				pH				pH				pH			
	5	6	7	8	5	6	7	8	5	6	7	8	5	6	7	8
*E. coli* 33504	100				>100				>100				100		50	
*E. coli* 32313	>100		100		>100				>100				>100			50
*K. pneumoniae* 33443	>100				>100				>100				>100			
*K. pneumoniae* 33163	>100				>100				>100				>100			
*P. mirabilis* 33877	>100				>100				>100				>100			
*P. mirabilis* 32470	>100				>100				>100				>100			

**Table 2:** MICs for PAβN, CCCP, and V9302 on *E. coli*, *K. pneumoniae*, and *P. mirabilis* strains.

MIC (µM)	PAβN				CCCP				V9302			
	pH				pH				pH			
	5	6	7	8	5	6	7	8	5	6	7	8
*E. coli* 33504	>100				12.5	6.25	25	100	100	50		>100
*E. coli* 32313	>100				12.5		25	100	100			>100
*K. pneumoniae* 33443	>100				25	>100			>100			
*K. pneumoniae* 33163	100	>100			100	>100			>100			
*P. mirabilis* 33877	>100				6.25	6.25	12.5	25	>100		50	>100
*P. mirabilis* 32470	>100				25				>100		100	>100

PAβN: phenylalanine-arginine β-naphthylamide, CCCP: carbonyl cyanide m-chlorophenyl hydrazone.

**Table 3:** Effect of selected compounds on the MIC values of antibiotics against *K. pneumoniae* strains.

MIC (µg/mL)	*K. pneumoniae* 33443			
	pH			
	5	6	7	8
CIP	100	100	25	6.25
CIP + TZ	ND	ND	12.5	ND
CIP + PMZ	ND	ND	12.5	ND
CIP + PAβN	100	25	1.56	0.39
FO	50	100	100	100
FO + PAβN	50	100	100	50
**MIC (µg/mL)**	***K. pneumoniae* 33163**			
	**pH**			
	**5**	**6**	**7**	**8**
CIP	>100	>100	>100	100
CIP + PAβN	>100	>100	>100	50

CIP: ciprofloxacin, TZ: thioridazine, PMZ: promethazine, PAβN: phenylalanine-arginine β-naphthylamide, FO: fosfomycin tromethamine, ND: not determined.

## Data Availability

This data presented in this study is available on request from the corresponding author.

## Supplementary Materials

The following supporting information can be downloaded at: https://www.mdpi.com/article/10.3390/antibiotics14100988/s1, Table S1: 95% confidence intervals (CIs) of biofilm inhibition (%) exerted by the EPI compounds on the bacteria.
