# Supplementary material for: Repurposed Drugs and Efflux Pump Inhibitors Against Gram-Negative Urinary Tract Pathogenic Bacteria
Source: Antibiotics (Basel). 2025 Oct 2;14(10):988. doi: 10.3390/antibiotics14100988 (PMC12561020; doi:10.3390/antibiotics14100988)
Supplement: Supplementary file 1 [file antibiotics-14-00988-s001.zip › antibiotics-3877291-supplementary.pdf]

Repurposed drugs and efflux pump inhibitors against Gram-negative urinary tract pathogenic bacteria

Annamária Kincses [1,2], Márta Nové [1], Jina Asefi [1] and Gabriella Spengler [1,*]

[1] Department of Medical Microbiology, Albert Szent-Györgyi Health Center and Albert Szent-Györgyi Medical School, University of Szeged, Semmelweis utca 6, 6725 Szeged, Hungary; kincses.annamaria90@gmail.com (A.K.); bozoki-nove.marta@med.u-szeged.hu (M.N.); jina.asefi@yahoo.de (J.A.); spengler.gabriella@med.u-szeged.hu (G.S)
[2] Institute of Pharmacognosy, Faculty of Pharmacy, University of Szeged, Eötvös utca 6, 6720 Szeged, Hungary; kincses.annamaria90@gmail.com
* Correspondence: spengler.gabriella@med.u-szeged.hu

SUPPLEMENTARY INFORMATION

Table S1. 95% confidence intervals (CIs) of biofilm inhibition (%) exerted by the EPI compounds on the bacteria.* TZ: thioridazine, PMZ: promethazine, Fx: fluoxetine, Sr: sertraline, PAβN: phenylalanine-arginine β-naphthylamide, CCCP: carbonyl cyanide m-chlorophenyl hydrazone; ND: not determined.

**95% CI (%)**	***K. pneumoniae* 33443**			
	**pH**			
	**5**	**6**	**7**	**8**
TZ	32.67 to 41.27	38.88 to 41.12	59.90 to 63.12	48.43 to 51.07
PMZ	ND	ND	48.03 to 50.07	ND
Fx	ND	ND	36.49 to 38.27	ND
Sr	ND	21.52 to 30.23	38.90 to 43.18	37.58 to 40.12
PAβN	48.69 to 55.59	41.17 to 45.05	50.51 to 60.95	39.63 to 42.51
CCCP	-24.20 to -13.62	ND	ND	-38.28 to -32.36
V9302	54.90 to 61.96	47.34 to 51.66	59.52 to 74.32	58.60 to 61.14
**95% CI (%)**	***K. pneumoniae* 33163**			
	**pH**			
	**5**	**6**	**7**	**8**
TZ	ND	ND	42.06 to 45.04	ND
Fx	ND	ND	25.29 to 28.27	ND
PAβN	50.23 to 63.19	49.09 to 55.59	68.40 to 72.22	44.95 to 48.47
V9302	55.45 to 59.27	56.97 to 61.83	70.12 to 73.64	45.22 to 49.20
**95% CI (%)**	***P. mirabilis* 33877**			
	**pH**			
	**5**	**6**	**7**	**8**
Fx	ND	ND	ND	33.56 to 46.28
PAβN	ND	ND	ND	22.49 to 26.47
CCCP	ND	15.60 to 21.90	-33.43 to -16.49	ND
V9302	ND	ND	ND	34.60 to 37.94

95% CI (%)	*P. mirabilis* 32470			
	pH			
	5	6	7	8
TZ	26.64 to 30.62	ND	13.22 to 17.52	ND
Fx	16.96 to 20.14	ND	ND	ND
Sr	ND	ND	11.17 to 13.05	ND
PAβN	ND	ND	24.44 to 27.72	ND
CCCP	28.85 to 33.24	ND	-38.68 to -32.18	ND
V9302	ND	ND	13.22 to 15.60	15.20 to 21.96

95% CI (%)	*E. coli* 32313			
	pH			
	5	6	7	8
PMZ	ND	ND	-20.05 to -9.08	ND
V9302	ND	54.42 to 56.80	ND	ND

*CIs were calculated only for statistically significant results.
